# Computer-assisted client assessment survey for mental health: patient and health provider perspectives

**DOI:** 10.1186/s12913-016-1756-0

**Published:** 2016-09-23

**Authors:** Manuela Ferrari, Farah Ahmad, Yogendra Shakya, Cliff Ledwos, Kwame McKenzie

**Affiliations:** 1 School of Health Policy and Management, York University, 4700 Keele Street, HNES Building, Rm 414, Toronto, ON M3J1P3 Canada; 2 Access Alliance Multicultural Health and Community Services, 340 College Street, Suite 500, Toronto, ON M5T3A9 Canada; 3 Wellesley Institute, Centre for Addiction & Mental Health, 33 Russell Street, Toronto, ON M5S2S1 Canada

**Keywords:** Computer-assisted, Mental health assessment, Community health centre, Mixed-method research, Canada

## Abstract

**Background:**

The worldwide rise in common mental disorders (CMDs) is posing challenges in the provision of and access to care, particularly for immigrant, refugee and racialized groups from low-income backgrounds. eHealth tools, such as the Interactive Computer-Assisted Client Assessment Survey (iCCAS) may reduce some barriers to access. iCCAS is a tablet-based, touch-screen self-assessment completed by clients while waiting to see their family physician (FP) or nurse practitioner (NP). In an academic-community initiative, iCCAS was made available in English and Spanish at a Community Health Centre in Toronto through a mixed-method trial.

**Methods:**

This paper reports the perspectives of clients in the iCCAS group (*n* = 74) collected through an exit survey, and the perspectives of 9 providers (four FP and five NP) gathered through qualitative interviews. Client acceptance of the tool was assessed for cognitive and technical dimensions of their experience. They rated twelve items for perceived *Benefits* and *Barriers* and four questions for the technical quality.

**Results:**

Most clients reported that the iCCAS completion time was acceptable (94.5 %), the touch-screen was easy to use (97.3 %), and the instructions (93.2 %) and questions (94.6 %) were clear. Clients endorsed the tool’s *Benefits*, but were unsure about *Barriers* to information privacy and provider interaction (mean 4.1, 2.6 and 2.8, respectively on a five-point scale). Qualitative analysis of the provider interviews identified five themes: *challenges in Assessing Mental Health Services*, such as case complexity, time, language and stigma; the *Tool*’*s Benefits*, including non-intrusive prompting of clients to discuss mental health, and facilitation of providers’ assessment and care plans; the *Tool*’*s Integration* into everyday practice; *Challenges for Use* (e.g. time); and *Promoting Integration Effectively*, centered on the timing of screening, setting readiness, language diversity, and technological advances.

**Conclusions:**

Participant clients and providers perceived iCCAS as an easy and useful tool for mental health assessments at the Community Health Centre and similar settings. The findings are anticipated to inform further work in this area.

**Trial registration:**

ClinicalTrials.gov; NCT02023957; Registered retrospectively 12 Dec. 2013

## Background

Common mental disorders (CMDs) such as depression, anxiety, alcohol abuse, and post-traumatic issues are on the rise globally. The World Health Organization posits that depression will rank first in the national burden of diseases in low income, middle and high income countries by 2030 [1]. Indeed, CMDs have recently emerged as a priority area in several countries including Canada [2]. According to the Mental Health Commission of Canada, more than six million individuals are living with a mental illness [2]. These conditions have significant impacts on individuals, their families, and society. The direct cost of mental illnesses to the Canadian economy is estimated at more than $51 billion annually [3, 4]. However, effective treatments are available for most CMDs, including those noted above [2, 5].

The burden of CMDs is not evenly distributed across the population. Immigrant, refugee, ethnically marginalized, and low-income groups are identified as vulnerable communities [6, 7]. For immigrants and refugees, the social context of migration and settlement in a new country is often fraught with challenges and discrimination, such as in securing suitable employment and housing. These challenges are some of the many social determinants that increase the vulnerability of immigrants and refugees to mental health issues [8–10]. In addition, these groups experience barriers to accessing timely care, such as a lack of knowledge of available services, difficulties with language and communications, and stigma associated with mental health problems and the use of services [7–10].

A window of opportunity exists through Community Health Centres to address mental health inequities. Canada has a universal health insurance system in which Community Health Centres play a vital role in the provision of primary care. A key mandate of the Centres is to reach vulnerable and disadvantaged groups-including low-income immigrants, refugees and racialized families-through integrated and interdisciplinary clinical care provided alongside community engagement and outreach [11]. Community Health Centres recognize the impact that social determinants of health have on the populations they serve. Their interdisciplinary teams include family physicians, nurse practitioners, registered nurses, social workers, dieticians, settlement workers, interpreters, and community outreach peers. Studies in Ontario show that Community Health Centres serve a higher number of individuals with serious mental illnesses than other primary care models (e.g., solo family physicians and Family Health Teams) in both urban and rural areas [11]. The Centres also appear to perform better in chronic disease management, as indicated by lower numbers of emergency visits by their clients compared to those enrolled in other primary care models [11–17]. Yet Community Health Centres are under resource strain due to the system level issues (e.g. growing needs of aging population) while the populations they serve face multiple socio-economic vulnerabilities and complex health needs [11]. Interactive eHealth tools, tailored to screen for complex health issues and risks faced by low-income immigrants, refugees and racialized groups, could assist Community Health Centres in addressing growing demands of both the community and the healthcare system.

### Interactive eHealth tools, mental health & primary care

The use of computer-assisted screening and history-taking for mental health can be traced back to the 1960s [18, 19]. Since then, this area has seen tremendous growth resulting from advancements in technologies. Several studies document patient comfort and ease in completing questionnaires through computers for socially sensitive health risks (e.g. sexual, alcohol, drug, HIV and violence related behaviour) when compared to in-person interviews [20–24]. Others report similarity in the sensitivity and specificity of CMD scales administered by computers as compared to clinicians [25, 26].

New digital technologies are further expanding the scope of screening and history-taking by transcending communication barriers between clinician and patient [27]. The first generation of digital tools focused either on clinicians (e.g. hand-held decision trees) [28–30] or on patients (e.g. standalone self-assessments) [31]. The recent generation of tools is attempting to employ “dual engagement”, engaging patient and provider simultaneously in order to overcome a conceptual deficit wherein the results of assessment reports were generated automatically for clinicians only. This opportunity of dual engagement is recognized as an important development for addressing mental health concerns in a timely and sensitive manner [32, 33].

We describe here some of the studies that align with the overarching aims of this project, namely, testing of dual engagement tools in primary care settings with a focus on depression: Electronic Case-finding and Help Assessment Tool (eCHAT) in New Zealand, Promote Health in Canada, and My Own Health Report (MOHR) in the United States [34–36]. Goodyear-Smith et al. report on eCHAT, a screening tool that identified anxiety, depression, physical activity and smoking as priority areas for patient assistance [34]. The tool had 20 questions (two questions for depression, one for anxiety, and two for alcohol) and was completed by patients (84 % of those invited) in clinician waiting rooms using iPads. eCHAT generated a single report for the clinician and patient for review during the consult. In a randomized controlled trial Ahmad et al. examined Promote Health, a touch-screen, multi-risk survey completed by female patients in a family practice clinic [35]. The survey had 79 questions (nine on partner violence, eight on depression, and four on alcohol use) from validated scales, and the tool produced plain language reports for patients and separate summaries of results for clinicians. The authors found that the tool doubled the odds of discussion and detection of partner violence and mental health symptoms in the intervention group compared to usual care. Krist et al. examined MOHR, electronic or paper-based, for assessment and identification of unhealthy behaviors and CMDs in a pragmatic cluster trial with nine diverse primary care clinics in urban and rural areas [36]. The tool asked 23 questions using scales of Patient Health Questionnaire-9 (PHQ-9), Generalized Anxiety Disorder-7 (GAD-7), Alcohol Use Disorder Identification Test-C (AUDIT-C) and Drug Abuse Screening Test-10 (DAST-10) for depression, anxiety, alcohol and drug abuse, and generated two distinct types of summary reports for patients and clinicians. Using the RE-AIM (reach, effectiveness, adoption, implementation and maintenance) evaluation framework, the authors concluded that primary care practices can make use of CMDs assessments and health behavior tools when counseling resources are available to these clinics.

Based on these findings, it can be expected that primary care settings comprising multidisciplinary teams (e.g. Community Health Centres) and serving vulnerable and disadvantaged groups-including low-income immigrants, refugees and racialized families-with risks of experiencing CMDs could especially benefit from eHealth tools. Yet, scholarly work on interactive health-risk assessments for Community Health Centres is in its infancy. An exception is the work by Ahmad and colleagues in developing and piloting a Computer-assisted Psychosocial Risk Assessment (CaPRA) survey for Dari/Farsi-speaking Afghan refugees visiting Access Alliance in Toronto, Ontario. This touch-screen, self-assessment tool, which generated point-of-care reports for clients and providers, was effective in promoting intentions to seek help from a psychosocial counsellor (intervention 72 % vs. usual care 46 %) [37]. More recently, Ahmad and colleagues developed a tool in English and Spanish that focused on CMDs: Interactive Computer-Assisted Client Assessment Survey (iCCAS). The tool was evaluated in a pilot randomized controlled trial and doubled the odds of discussion and detection of mental health symptoms in the intervention group compared to usual care (under-review by CMAJ). The current paper reports the perspectives of clients and providers who participated in the trial.

## Methods

### Study setting

The study was conducted in partnership with a Toronto-based Community Health Centre, Access Alliance Multicultural Health and Community Services. Toronto is a metropolitan and culturally diverse city with a population of 2.6 million. Recent census results show that 51 % of Toronto residents are foreign-born compared to 20.6 % at the national level [38]. During the past 5 years, Asia (including the Middle East) was Canada’s largest source of immigrants, although the share of immigration from Africa, the Caribbean, Central and South America increased slightly [38]. In 2011, 90 % of Canada’s 6.8 million foreign-born individuals lived in the nation’s largest urban centers of Toronto, Montreal, Vancouver, and Calgary [38]. Given the multicultural composition of Toronto, Access Alliance serves a large number of immigrants, refugees, and low-income groups. This Centre offers both primary care and community services through multidisciplinary teams working at three sites across the city. During 2012–13, Access Alliance had 47,682 direct agency encounters. Out of the total encounters, 47 % were primary care and 20 % were for the newcomer’s resource center. A significant number of primary care encounters also involved a language interpreter [39].

### Ethics, consent and permissions

The study protocol was planned in collaboration with Access Alliance and research ethics approval was obtained from York University (certificate no: e2013–291). All participants provided informed written consent.

### Intervention

iCCAS consists of a tablet-based, touch-screen survey that is completed by clients during the waiting period before seeing their providers in a primary care setting. It was developed collaboratively by an academic-community team using a participatory approach. The process began with a comprehensive literature review on CMD and their assessment scales. The team then employed a criteria-based matrix to decide how many questions and issues should be included in the tool without overburdening the patient and/or the provider. The criteria were: scale length, reliability, validity, use in diverse groups, use in e-health tools, use in primary care settings, and association with depression. The team settled on 52-items for iCCAS, including validated screening scales for major depression, generalized anxiety disorder, post-traumatic stress disorder and harmful drinking along with social determinants of mental health (e.g., housing, employment status, immigrant status). Specific CMD scales are the: PHQ-9 [40]; GAD-7 [41]; 4-item CAGE (cut-annoyed-guilty-eye) [42]; and 4-item Post Traumatic Stress Disorder-Primary Care (PTSD-PC) [43] scale. Following completion of the survey, the program generates individualized reports for providers and clients at the point-of-care. The clinician report comprises a brief risk-summary with possible referrals and community contacts. The client report comprises a plain-language recommendation sheet for the identified risks, with suggestions about available services and encouragements to seek the advice of the attending clinician. Because Spanish-speaking patients are a major client group at the partnering CHC, the iCCAS survey and client messages were developed in Spanish through a translation and back-translation process; any discrepancies were resolved through discussion and comparison with available Spanish versions (e.g., http://www.coloradohealthpartnerships.com).

A usability study was conducted with seven patients (four English, three Spanish) and five providers (all English). Their suggestions related to formatting (e.g. font and arrows), difficult words (e.g. transgender, stable housing, common-law, qualified health professional, appetite, concentrate and nightmares), additional contacts (e.g. emergency shelters), and description of score ranges in the clinician report. Corresponding changes were made, including examples in parentheses for difficult words, and the prototype was refined.

### Design and procedures

The parent study evaluated the intervention impact by conducting a randomized controlled trial (RCT). Prior to the RCT, all family physicians (FP) and nurse providers (NP) at the partnering Centre provided informed consent and attended a 1.5-h workshop on the four CMDs for which the iCCAS tool screens. Providers also discussed various scenarios of seeing patients with a positive-screen for one or more CMDs detected through iCCAS. All FPs and NPs agreed to assess individual patient safety and severity of symptoms to inform care plans, such as an immediate comprehensive mental health assessment or a follow-up visit if preferred by a patient. Adult clients seeing a FP or NP were eligible if they could speak and read English or Spanish. Three research assistants (two bilingual and one English speaking) approached potential client-participants in the waiting room, applied eligibility criteria and obtained informed consent in private (response rate of 78.6 %). Consenting clients were then randomly assigned to the iCCAS group (intervention) or the usual care group (control). Each client in the intervention group completed the iCCAS and received a computer-generated report. The clinician received a report attached to the patient’s medical chart. Each client in the control group received usual care with no risk assessment before the consultation. Clients in both groups completed a paper-and-pencil Exit Survey after the visit; they received a $30 honorarium and a list of community-based services. This paper reports the clients’ perspectives measured quantitatively through post-intervention Exit Surveys, and perspectives of providers gathered through qualitative interviews after the trial.

### Data collection

The Exit Survey collected information on clients’ demographics along with cognitive and technical dimensions of the tool’s acceptance (see Table 1). The cognitive dimensions of acceptance were assessed for perceived *Benefits* (6 items), *Privacy*-*Barriers* (3 items), and *Interaction*-*Barriers* (3 items) when using a touch-screen computer device to complete a self-administered, client health-risk survey with tailored reports at the point-of-care for providers and clients [44, 45]. Each item was rated for level of agreement or disagreement on a 5-point scale (strongly disagree =1, disagree =2, not sure =3, agree =4, strongly agree =5). Examples of items for perceived *Benefits* included: ‘the computer is a good way to ask about social and emotional issues’; ‘I would feel comfortable answering questions on a computer’; and ‘it would save the provider’s time’. Examples of items for perceived *Privacy*-*Barriers* are: ‘I would worry about confidentiality when completing a computer survey’; and ‘too many mistakes will be made’. Examples of items for perceived *Interaction*-*Barriers* included: ‘providers would spend less time with patients’; and ‘there will be loss of personal communication with a provider’. The technical dimensions of acceptance were measured by four Quality Assessment questions [46] concerning the comfort level of using the touch-screen, ease of following the survey instructions and of reading the onscreen questions. Items were rated on a 4-point scale (very easy =1, easy =2, difficult =3, very difficult =4). An additional question was included on the acceptability of *time* for survey completion (yes/no). Participants were also invited to provide open-ended comments.

**Table 1:** Perspectives of Participating Clients (*n* = 74)

Computerized Lifestyle Assessment Scale mean (standard deviation)	
*Perceived Benefits*, *overall mean 4.1* (*0.7*)	
1. I would feel comfortable answering questions on a computer.	4.3 (0.8)
2. The computer is a good way to ask about social and emotional issues.	4.3 (0.7)
3. It would save the provider’s time.	4.1 (0.9)
4. Computer-assisted risk assessment will help providers with questions on social and emotional health.	4.0 (0.8)
5. Providers will make better health assessments with such computer systems.	3.9 (0.9)
6. Computer-assisted health risk assessment can be trusted.	3.9 (0.9)
*Perceived Privacy*-*Barriers*, *overall mean 2.6* (*0.8*)	
1. I would worry about confidentiality when completing computer survey.	2.9 (1.2)
2. I do not want certain information about me on computer.	2.7 (1.1)
3. Too many mistakes will be made with the computer-assisted risk assessment.	2.3 (0.9)
*Perceived Interaction*-*Barriers*, *overall mean 2.8* (*0.8*)	
1. Providers would spend less time with patients.	3.2 (1.2)
2. There will be loss of personal communication with a provider.	2.8 (1.1)
3. I would find another provider with no such tool.	2.4 (1.0)
Quality Assessment, count (percentage)	
*Using the touch screen*	
Very Easy	62 (83.8)
Easy	10 (13.5)
Difficult/Very Difficult	2 (2.8)
*Following the survey instruction, count (percentage)*	
Very Easy	51 (68.9)
Easy	18 (24.3)
Difficult/Very Difficult	5 (6.8)
*Reading the questions on screen, count (percentage)*	
Very Easy	61 (82.4)
Easy	9 (12.2)
Difficult/Very Difficult	4 (5.4)
*Acceptable time for survey completion, count (percentage)*	
Yes	70 (94.6)
No	4 (5.4)

In addition to the above, we conducted semi-structured, in-person interviews with the participating FPs and NPs. A trained research assistant, hired for the purpose of this study, conducted all interviews using an interview guide with open-ended questions. The guide covered the following topics: assessment of common mental health conditions by FPs or NPs in a day-to-day practice; overall experience with the iCCAS tool; barriers/facilitators for health providers and clients in using the tool; and recommendations for future use. The interviewer also completed field notes at the end of each interview. The interview length varied from twenty to forty minutes. All interviews were held at a time and place convenient to the participating providers.

### Analysis

The quantitative data gathered through the Exit Survey was analyzed using Statistical Package for Social Sciences (SPSS version 18). We executed descriptive statistics (frequencies, proportions, and means) and examined scores of perceived *Benefits* and *Barriers* by socio-demographic characteristics (student *t*-test and ANOVA). The scale items were reverse coded prior to analyses so that a score of one referred to ‘strongly disagree’ (i.e. low benefits or low barriers) and five to ‘strongly agree’ (i.e. high benefits or high barriers). Some response categories were collapsed due to small sample size (e.g., use of computers). All qualitative interviews were digitally recorded and transcribed verbatim. Transcriptions were organized using N-Vivo software (N-Vivo version 10). Transcriptions were then analyzed according to Bauer and Clark’s steps of thematic analysis [47]. A team member (M.F.) first read and reread all of the transcribed data and developed a preliminary coding scheme. This was refined by independent reading of the transcribed data by another team member (F.A.). Members made use of an inductive approach, rather than a theoretically driven one, so that the identified themes were strongly linked to the data themselves. Several strategies were used for trustworthiness in the qualitative findings [48]. For *credibility* and *authenticity*, the team focused on rigour in design, data collation, and analysis. For *criticality* and *integrity*, the team used field notes and team debriefing during data collection.

## Results

### Participants

Seventy-four clients (male 25; female 49) with a mean age of 36.6 years (standard deviation, SD 12.8) completed the intervention. Fifty-eight clients completed iCCAS in English (78.4 %) and 16 in Spanish (21.6 %). Almost all of the participant clients were immigrants and reported their English language skills as ‘good’ on a 5-point scale with a mean of 3.1 (SD 1.0). Thirty-seven percent had college or university education and only 25 % reported having full- or part-time employment. Fifty percent reported using computers every day. Overall, participants rated their self-perceived health as ‘fair’ on a 5-point scale with a mean of 2.84 (SD 1.0).

The face-to-face interviews were conducted with the nine providers who participated in the trial. This included four FP and five NP (male 4; female 5) with an age range of 20s to over 40. To maintain providers’ confidentiality, their demographic information is not included here.

The following section provides patient perspectives assessed quantitatively, and perspectives of providers identified through thematic analysis.

### Client perspectives

Analysis of the perceived *Benefits* and *Barriers* sub-scales showed that, overall, clients had positive attitudes towards iCCAS (Table 1). On a scale of 1 to 5, clients ‘agreed’ with the perceived *Benefits* of the tool (mean = 4.08). Client scores for the perceived *Privacy*-*Barriers* (mean = 2.63) and *Interaction*-*Barriers* (mean = 2.81) indicate ‘not sure’ status. The *t*-test and ANOVA analyses showed that the scores for each of the three sub-scales did not differ statistically by demographic characteristics of the clients including: age; gender; education; income; citizenship/resident versus other status in Canada; years lived in Canada; and overall self-rated health.

The analysis of the Quality Assessment questions showed clients’ satisfaction with the technological and literacy elements of iCCAS. Most clients reported iCCAS completion time was acceptable (94.5 %), touch screen was easy to use (97.3 %), instructions were clear (93.2 %) and questions were clear (94.6 %). Seven clients reported some difficulty and specified the reasons as wording or grammar of questions and structured nature of the survey; one had technical difficulty in clicking the screen. Out of these seven clients, most were recent immigrants (*n* = 6); women (*n* = 5) and identified ‘poor’ or ‘fair’ English or Spanish language abilities (*n* = 5) though they reported using computers everyday (*n* = 6).

Ten clients provided open-ended comments on how to improve the iCCAS application; these were analyzed by two authors for overarching themes. Four reinforced their positive experience with iCCAS (e.g., “*So far everything was absolutely good*”). Others suggested: extending the application to android; decreasing the number of questions and improving the clarity; and adding more information about diagnosis.

### Provider perspectives

Five themes were identified through thematic analysis of the qualitative interviews with providers: (a) challenges in assessing mental health; (b) benefits of using iCCAS; (c) challenges of using iCCAS; (d) providers’ interests in integrating iCCAS into everyday practices; and (e) promoting an effective integration of iCCAS into primary care practices. Each theme builds on sub-themes which are reported in Table 2 and described within the next section. For transparency and ease of interpretation per qualitative methodology, major theme and sub-theme frequencies are identified by using descriptors whereby *a few providers* refers to less than half of the participants, *the majority of* or *several providers* refers to more than half. In other instances we provide exact numbers, such as *one provider* or *all*. Within the qualitative data analysis process, we compared and contrasted FP interviews versus NP interviews and did not find any major differences.

- Providers’ challenges in assessing mental health
  All of the participant providers from the community health center perceived high prevalence of mental health issues among their clients but noted that assessment of mental health in routine practice is challenging due to the high complexity of clients who present with a number of health conditions at the same time. In the words of one participant:
  I’ve been here long enough to realize the prevalence of underlying mental health conditions… I almost have an assumption that there are mental health issues unless I’ve found otherwise. There’s the whole stress [of] migration but [I] do not denounce the various degrees of resilience in the population (FP#1).
  All providers associated their clients’ vulnerability with their socio-economic circumstances (e.g., migrations, housing, language, and employment issues). Often, participant providers found it necessary to prioritize which of a client’s conditions to address first. A few providers pointed to frequent visits due to somatic complaints.
  We have frequent utilizers of our services, … we see numerous appointments for somatic complaints that don’t have any readily [available] explanation [of] the vague symptoms (FP#1)
  So often with our patients, especially with the complexity of other issues, we need to deal with multiple issues at each visit, and so as we all know mental health takes a lot of time to—to even screen properly, diagnose, and assess. So it’s often hard to find the time to be able to do that in routine visits. (FP#6)
  As just described in the last quote, along with the clinical complexity of the cases, the lack of time to properly discuss mental health problems with clients was reported as a barrier by several interviewed providers.
  They are usually coming in presenting with a different issue; then the mental health issues are sort of teased out, or come up, during the interview. You don’t really have much time to address them. Because they may come in thinking they have a pain in their foot and then it end up having something else. So, time constraints. (FP#4)
  Even if language interpreters were regularly available (either in person or over the phone) during clinical visits, language barriers and communication difficulties were reported as major challenges by the participant FP and NP in assessing common mental health conditions. In the words of one provider, “language can be a huge barrier, we try to use interpreters whenever they come but still things get lost in translation a little bit” (FP#2). The assistance from interpreters also added time to the clinical encounters. Others described discomfort of some clients in the presence of interpreters due to the sensitivity of topics under discussion. Furthermore, a provider described that female clients often do not feel comfortable expressing their feelings, issues, and health status to a male interpreter. According to a few providers interviewed, clients often feared that interpreters, belonging to the same community, would share with others the content of their clinical conversations.
  The interpreters, they have the skills and they’ve done the training but they are from the same community. … [Patients worry] that message will be carried back to their community … Even though, privacy laws [exist]… They know that whatever they say is actually not just being said through the interpreter but is being said to [the person] also. There are all these little intricacies when you think of somebody who is not English speaking being able to tell you how they feel. (NP#3)
  All providers identified mental health stigma as a major barrier to clients disclosing mental health issues and/or discussing their symptoms. They also felt that the level of stigma varied across ethno-cultural groups and social status of their clients. A few providers pointed to variations in symptom presentation or to the tendencies of certain cultural groups to view any mental health issues in a highly negative light. In a few instances, providers reported that certain cultural groups may deny or ignore mental health problems. This demanded, in their views, cultural sensitivity in counselling.
  There is a big stigma attached to mental illness so even if the client themselves wants to disclose how they feel the relative that has brought them, because they are feeling so low and so down, [They] says “Oh it’s not so bad.” (NP#3)
  [this is a] vast over-generalization, but a lot of the sub-Saharan African countries I find don’t have this culture. It’s like…mental health doesn’t exist. A lot of the South Asian communities as well, so especially like our North Koreans and our Bhutanese population as well. … Some people have very negative views on it. (FP#3)
  Despite the many and unique challenges that participant providers were facing in assessing their clients’ mental health, they acknowledged that they were working in an interdisciplinary primary care setting which gives strong emphasis to mental health issues and treatment.
- Perceived benefits of using iCCAS
  Participant providers described several benefits of using iCCAS. Those benefits were directly related to clients’ well-being and self-assessment as well as to providers’ clinical practices.
  *Perceived benefits to clients*
  All participant providers appreciated that clients in the iCCAS group were able to assess their mental health status independently. This self-awareness was recognized as key in a client’s ability to bring up personal mental health issues during clinical visits.
  I guess it’s to put the person in the head space to get them thinking about their mental status, because they already have an agenda in their head when they first arrive. (NP#3)
  It’s good for patients that would want to sort of self-manage or start things on their own. (NP#6)
  Several providers reported that iCCAS offered a non-invasive way to assess mental health problems. In relation to the anonymous and non-judgmental interaction possible through computer-based assessment, they perceived that clients might have felt less social pressure and stigma, which then enhanced their comfort with honestly reporting emotions and thoughts.
  Sometimes it’s easier to talk to a computer that is “blank.” Then they can say exactly what they want to say. If they are in front of a person then no matter how blank you keep your face, which you never do, you always try to be pleasant. … Where as to a computer, if you’re feeling low, they can be low, there’s no expectation of how they are supposed to behave. So, I think it’s easier to express how you feel, although, there is something to be said with empathy, so that you can draw things out. But certainly as a starting tool, for screening … (NP#3)
  iCCAS was also perceived as an effective self-assessment tool to make mental health issues more tangible and accessible for clients to begin the process of recognizing, making sense of, questioning and/or disclosing possible symptoms or issues. A few providers used the term ‘*normalize*’ mental health issues to describe these processes. One provider described how iCCAS screening seemed to have empowered a few clients by providing them with tangible proof of their distress and mental health issues.
  I think at least in a few instances … it did have a positive influence on the patients. It was almost another affirmation that they were having issues and something else to speak to, not necessarily to provide diagnosis or treatment, but a recognition. (FP#1)
  Participant providers reinforced the importance of clients receiving preliminary feedback, suggestions for next steps, and resources via the iCCAS survey and client recommendation sheet. This enhanced clients’ confidence and ‘*gave permission to speak about that*’. Such resources are generally provided to clients during the first intake meeting, described providers, but over time clients may forget or not become aware of new resources which become available.
  Maybe it was a matter of their confidence being bolstered by the fact that they have something there that was an objective measure. They could show me….and stimulated a few of them to address it at that appointment and allow me to address it more easily (FP#1)
  I think they are happy to have someone else [who] is willing to listen. At the same time, providing the resources that they probably need. Many people forget about what’s available … they don’t know what’s available out there. (NP#4)
  The fact that iCCAS was able to serve Spanish-speaking clients in their first language was also identified as a strength.
  *Perceived benefits to clinical practices*
  Participating providers described how the iCCAS application was an effective, user-friendly screening tool to assess mental health problems in primary care.
  I think it’s really good, I think that it brings a lot too as far as screening, I could definitely see value in—in like the general practice as far as having those questions asked for you so you’re sort of bringing in and it will trigger the discussions around it. [I] t’s user friendly and you know it’s used quickly to interpret the results. (NP#5)
  Several providers found the iCCAS report easy to review and effective in providing cues through clients’ mental health screening scores along with recommendations.
  I guess it was a little plain. It had titles. I did not have difficulty reading it. My eye was drawn towards different things. And the fact that suicidality was highlighted … it was a really good thing. It made me go “ohhh.” And I thought about our discussion and I was not worried about his safety. (FP#4)
  According to several participating providers, iCCAS provided the opportunity to initiate a discussion on CMDs during routine visits. More importantly, providers became aware of possible distress and/or problems by reading the iCCAS-generated clinician report and, if needed, they were able to ask specific questions to better assess a client’s well-being. In some instances, new mental health issues-particularly on sensitive issues like abuse-were identified through the iCCAS screening. With respect to this facilitative screening role of iCCAS, participating providers noted benefits for clients (more comprehensive ‘*care plan*’) as well as to providers/system (saving time).
  Absolutely. It definitely saved me time because a couple times things were flagged on that form that I didn’t previously know about or I wouldn’t have asked about based on the nature of our visit type that particular time. So, it is good to know because it did impact my care plan. So, I think it was beneficial but it is also something that I wouldn’t have otherwise known about because I probably wouldn’t have taken the time in that particular instance to identify it. (NP#1)
  It’s a screening tool. I did have one young lady who had potentially a problem with abuse in the home and that was pulled out. I was very grateful for that and another woman who was suffering from a lot of stress that was bringing her down to a low grade depression. I remember those clients. And I remember the little piece of paper but the one with the abuse had at the bottom of it all the links to where they could call. (NP#3)
- Perceived challenges in using iCCAS
  Several providers recognized time as a constant challenge, particularly for the clients who came for a very different reason and were offered mental health assessment.
  To be honest I think a lot of the time the patient came in for a totally different reason, like knee pain or something like that and so they want to talk about the knee pain and by the time the appointment was over there was no time to talk about the iCCAS study. (FP#2)
  A few providers reported having some false alarms through the self-screen report. Providers identified these through discussions with clients, who didn’t realize that screening questions (e.g. PHQ-9 and GAD-7) were for symptoms experienced only during the last 2 weeks. According to some, language or literacy level could have been the source for the discrepancies.
  There were a couple instances where the severity was a lot higher than I had anticipated and I queried the patient after the fact … in a couple instances they may have misinterpreted … they seemed to have answered the questions in terms of “in the past have you had this level of depression” … they weren’t necessarily relating it to the most recently [2 week period]… they were just answering about their worst experiences. (FP#1)
- Interest in integrating iCCAS into everyday practices
  In balancing the pros and cons of using iCCAS during routine clinical visits, all providers recognized its potential and confirmed that they would support its integration into regular clinical practices at the center. In the words of one provider, “I would be very supportive of it. I think it’s a very helpful tool”. (NP#3)
  All providers reported that iCCAS screening did not interfere with their clinical practices. As described, the screening tool positively impacted providers’ abilities to assess their client’s mental health. Although the length of visits may have been affected, providers mentioned that this was not a major concern and could be accommodated.
  No [it did not interfere with the visit], I think the only thing was time. As long as the patient was ok with starting the visit earlier with you and ending a little bit later with our visit. I think that it generally was well received from their perspective. I mean, I’m usually on time with my appointments and I never felt I had to wait for very long. Maybe a couple minutes. But it didn’t impact the flow of any of my appointments any further. (NP#1)
  A few commented that iCCAS helped with note-taking. Providers reported that, even if visit times did not allow the possibility to fully discuss the outcome of the iCCAS screening, the screening scores and overall feedback during the visit-which they kept on file-would be useful for discussions during follow-up visits.
- Promoting an effective integration of iCCAS into primary care practices
  To improve the technology for effective integration, providers made suggestions related to adaptations, integration, time of the screening, and settings. In relation to adaptation, the majority of providers expressed strong desire for a multi-language version of the tool. “I wish it was available in more languages so that we can access more patients” (NP#1). A few providers made suggestions about furthering the user-friendliness and clarity of the survey questions, such as addition of a preamble before sensitive questions, and simpler phrases for clients with lower levels of literacy. Several providers recognized the potential for iCCAS to support screening clients in different languages. In relation to integration, a few providers described the benefit of linking the iCCAS report with the electronic medical record (EMR) system in use at the center to optimize its accessibility, efficacy and creation of a database to inform practice.
  So, you know, the report itself goes into the EMR as opposed to the desk so it becomes part of the person’s chart. And if we wanted to do it on a regular basis. So, for a screening, say someone tests positive for things, then it could … alright, let’s get this person to do it again in 6 months’ time. (FP#4)
  In relation to the optimal time to use iCCAS, a few providers suggested that the potential benefits of iCCAS could be maximized if it was implemented during initial visits as well as during yearly wellness assessments.
  I am just trying to think of whether there would be a more optimal implementation time or period. We used to have an hour for initial appointments with our clients and you used to be able to flesh out some of those other mental health issues. It would need to be incorporated where there was an initial say 15 minutes at the beginning … an extended appointment perhaps to implement it … I don’t even know if it is realistic in this office. (FP#1)
  Actually I think that would be a very strong thing. [It] should actually be part of our routine, [once a year]. (FP#3)
  Given the high mental health needs of the population served by the Community Health Centre, providers described having a high level of vigilance for assessing the mental health of their clients. In this context, some providers reflected on stronger relevance of the tool for traditional general practice settings where mental health is not already a priority.
  We are so tuned in to mental health. It is a huge priority as an agency. This, although it is great and helpful and I like the concept, I think it would be much more of a benefit to an organization that is not so tuned in to mental health. (FP#4).

**Table 2:** Participating Providers’ Themes and Sub-Themes

Themes	Sub-Themes	Quotes
Providers’ challenges in assessing mental health	Complexity and severity of cases	One of the challenges, not only for mental health issues, but other chronic illnesses is that a large majority of my patients will only come to their appointments when they need something from me in particular. Not necessarily a medical issue. A lot of times it’s other issues related to filling out forms or seeking disability or stuff like that. So that can be a challenge…and a lot of times there is a lot to address in an appointment. (FP#1)
	Time	I would say the biggest reason is time. If a person doesn’t come with a complaint that might warrant that discussion, it tends not to be talked about. And for someone who is coming in with various episodic things, that could potentially not be addressed for a long time or ever. (NP#1)
	Language barriers	The biggest challenge would be in language. Because the way people present how they feel to the practitioner doesn’t necessarily reflect how they are feeling inside themselves. So, even if you have an interpreter, you’re not getting that nuance. (NP#3)
	Interpreters	When I’m having numerous patients back-to-back that require interpreters… it is not uncommon that they are scheduled like that instead of being interspersed with English-speaking clients where you can often make up some time. (FP#1)
	Mental health stigma	There’s definitely a stigma … Especially across cultures. It’s hard to really know from person to person and culture to culture because everyone [is] experiencing things differently. (NP#1)
	Vulnerable population	I think we have a big sort of burden of disease with mental health issues in the community health centre sector and ours as well. A lot of the clients that we see have more resistant or pervasive mental health issues, whether it’s post-traumatic stress disorder …we do have a lot of people who spend a lot of time waiting to come and be processed, to come to Canada as refugees (NP#5)
Perceived benefits of using iCCAS	Clients: self-awareness	I think [clients] appreciated it. I think for them, it was helping to unload a very big burden on them. So, I think it’s one more thing that took a little bit of the burden away. (NP#1) I think that the value of it in this setting is tuning people into their own mental health, showing people what resources are available, and, “Oh, why don’t you talk to this person?” and it also gets people to start thinking about their mental health. FP#4)
	Clients: disclosure	They felt more comfortable [talking about mental health] because they had already written it. They’ve already expressed it. Now they can build on what they had expressed. It wasn’t a new thought for them. It was very helpful. (NP#3)
	Clients: normalize	I also find that the last part that says *recommendations* has been really helpful too. Pretty much all of them say referral to social work, so I thought that that was really good because it [is] something that, normalizes it and it says that anyone can really benefit from this service so feel free to take advantage [of] …we’ve it available to you for free because it’s part of our organization and what we value. (NP#1)
	Clients: non-invasive	[The clients] entered all these symptoms and they think I have a problem rather than like a doctor telling you that you have a problem. (FP#2) For the provider point of view, there might be some things that come up because the iCCAS asked the question in a different way, or they are sitting there alone and their impulse is to answer in one way, while when they are in front of me their impulse is to answer another way. (FP#4)
	Clients: point-of-care feedback	It has the resources in it as well, so I think—it’s been a while since I saw one smokers’ helpline, there’s an alcohol one, there’s abuse, abuse, like, contact numbers for more information. (NP#5)
	Clinician: effective/efficient screening tool	I think it has a benefit, there are times when I’m dealing with the physical needs of the patient, but if I get the report it sort of alerts me to look into that part too… I don’t forget the mental part. (NP#2)
	Clinician: useful report	No, I think it’s quite clear. I usually only look at the left-hand side. That’s the main thing I look at. (NP#2)
	Clinician: identify new cases	The couple of times I had actually seen it was on a couple of people I was already managing their mental health issues. I think here mental health is very much front and centre. Both in the provider’s mind and also with the patients. It tends to come up more. So what the iCCAS report did for me was simply to solidify what was going on. Although there was one where it said that the person was feeling suicidal and I didn’t realize they were having those thoughts. So that would have been important. (FP#4)
Perceived challenges in using iCCAS	Time & many issues	Sometimes, not always, but often we will know about the mental health issues, or there’s other things that are pertinent to deal with at the time. (FP#3)
	Receiving iCCAS report	[The report] was sort of handed to me sometimes even in the middle of a visit, or when I had already started dealing with whatever issues. (FP#3)
	False alarm/ misinterpretation	There was an incident from iCCAS; it [the report] says “patient suffers severe depression and intention of hurting herself,” (…) but when I looked at the iCCAS report and I asked the patient … the patient goes “No, I’m fine, I don’t want to hurt myself or others.” I charted it too and I tried to follow up, the patient does have depression, but no intention of hurting themselves, so that was a little bit,… I don’t know what happened there. (NP#2)
Interest in integrating iCCAS into everyday practices.	Integration into regular practices	The more you can get yourself out there to discuss mental health, the better. So if there would be a way of being involved in the community’s services sector of Access Alliance for some of their programs or maybe not necessarily getting the entire group but getting a few people in the group that might be helpful, as well. (NP#1)
	iCCAS’s ability to promote better service	It would be a great way to advertise our community programs. That’s another thing, I sometimes find that the primary health care team and the community health program team are disconnected in a way… I find that I identify a lot of patients that could benefit from these programs and what I will do is, I’ll either write it on a piece of paper to say, “Hey, we have a community users desk at the front,” … but it just gets lost sometimes, sometimes that lady is just not there, a lot of that happens, maybe this is a good way. (FP#2)
Promoting an effective integration of iCCAS into primary care practices	Different languages	I think definitely to have it in other languages, and especially because our population…Yeah, like Farsi and Dari, like we have a lot of Afghan patients who again conceptually they don’t necessarily have the vocabulary around it. Korean… (FP#3)
	Integration with EMR	It’s good to incorporate with the computer system, the EMR system, also, it can be accessed though, by other clinicians, like a social worker can look at it. (NP#2)
	Time of the screening	I think it would be great for initial visits. If it can be timed with the initial visits, or pre-screening before people are seen at the clinic. … So if we knew that information before even seeing the patient, I think that would be very helpful as opposed to just dropping it in the middle of—of managing patients. (FP#3)
	Other primary care settings	The clinicians who work here, we all try very hard to stay on time. But, for example, the previous clinician he would see people and fit-ins and all of that. So that might of worked better for him. For us the—yeah the clinicians that were working during the iCCAS study are quite on time. … whereas at another practice maybe that wouldn’t be the case. And I think most doctors’ offices people don’t run on time until it might be easier to catch people when they’re in wait—in the waiting room. (FP#3)

## Discussion

Our study with healthcare providers and clients reveals general acceptance of and positive attitudes toward the studied interactive, touch screen-based self-assessment tool for common mental disorders. The majority of clients found the iCCAS tool easy to use with acceptable completion time, and agreed with the benefits of iCCAS in facilitating their care. Providers perceived that iCCAS enhanced their clinical practice, saved time, and helped clients in various ways. They also made suggestions about adapting and integrating iCCAS technology for future uptake and efficacy. The findings suggest that the tool has potential to reduce client and provider barriers in assessing and caring for CMDs in a timely and comprehensive manner. This is a step forward given the high mental health needs of clients, and the challenges facing providers, at Community Health Centres [49, 50]. We discuss the key findings further in relation to client activation, provider enablement, patient-centeredness and technological advances, which emerged across provider interviews and client surveys.

Provider perspectives on the helpfulness of the tool in enhancing client willingness to discuss mental health concerns emerged as a cross-cutting finding. Others have referred to such a phenomenon as “activated patients” (those who are knowledgeable about and are willing to discuss their risk status) and have found them to be effective “prompts” in medical visits for seeking preventive care and health promoting services [51–53]. According to providers in our study, the simple act of asking clients questions about their mental health status, using the self-administered process through touch-screen computer tablet, was helpful both in getting clients to self-assess their status and in making mental health issues more tangible and relatable, as described in the “perceived benefits to clients” section of the results. Providers also pointed to the non-intrusive nature of the tool, wherein clients completed the assessment independently, received individualized reports before the consult, and were able to review and reflect on these without feeling social pressure. Such processes could reduce mental health stigma, promoting timely disclosures and help-seeking. Patients in our study reported such support by agreeing with the tool’s utility in effectively asking about social and emotional issues and feeling comfortable in responding to such questions. Patient activation is an important step toward patient empowerment. Studies by scholars in New Zealand using a multi-risk eCHAT tool on lifestyle and mood disorders show that health problems are more likely to be treated when patients themselves first identify them, compared to when identification or discussion is initiated by a health care provider [54, 55]. Nonetheless, the degree of patient activation achieved through iCASS needs to be further examined for its value in supporting mental health detection and treatment in primary care settings that serve refugee and immigrant groups.

Provider enablement in the provision of care also emerged as a key cross-cutting point. On one side, providers perceived that iCCAS enhanced their clinical practice by consolidating their knowledge of existing mental health problems among their clients and by making them aware of new cases. On the other hand, participant clients agreed that the tool was helpful for providers as it allowed them to make better mental health assessments and to conserve their time. Another innovative operational feature of iCCAS was the generation of tailored reports for both clients and providers at the point of care. Other studies that have made use of such eHealth screening tools have reported operational feasibility and benefits to clients and providers, but the models lacked *tailored* reports for clients [28, 29, 56]. The iCCAS project advances the field through its focus on dual engagement. The findings suggest that the two types of reports generated by iCCAS led to not only activated patients but enhanced provider comfort in assessing sensitive issues and offering resources. At the same time, efforts around improving scale sensitivity-specificity should continue, as some providers noted a handful of false-positive cases. In future adaptations of the iCCAS tool, the 2 week timeframe for questions related to symptoms in the PHQ-9 and GAD-7 scales could be emphasized, possibly by using different font size and/or colour. Other scholars have noted that collection of patient information through computers could raise the likelihood of false-positive detections [20]. Yet computer-assisted interactive screening programs that concurrently provide advice to both clients and providers seem to have a greater likelihood of success as they increase vigilance by both parties [28]. Further developments are needed to advance interactive eHealth technologies for simultaneous engagement of providers and patients through feedback mechanisms. These initiatives could also draw from emerging work on patient portals (e.g. PatientSite) linked to EMR systems [57, 58].

Several of the benefits identified for iCCAS align with the widely-held goal of patient-centered primary care. Broadly, patient-centered care refers to the provision of care that is respectful of the client’s preferences and needs, that supports client-decision making, and that promotes timely access to care [59, 60]. The client’s ability to access their personal iCCAS report allowed them to gain self-awareness of their mental health status. Furthermore, clients had the opportunity to gain knowledge of self-help practices and/or treatment options, which supports client decision-making. Most importantly, as providers described, iCCAS opened a space to talk about clients’ mental health problems during clinical visits, and the possibility of initiating specialized care through referral to the clinic’s social worker and/or other specialized services. Nonetheless, some providers also pointed to an increase in the length of visits, which they managed by setting up follow-up visits. Future research should examine gains in patient-centeredness and its impact on patient outcomes. Our forthcoming paper on the randomized controlled trial with iCCAS would make a contribution in this area.

The study findings also provide directions for advancing the use of interactive eHealth technologies in primary care. For example, clients neither agreed nor disagreed regarding the tool’s impact on information privacy or on their personal interactions with providers. Possibly, provision of further details on these aspects (e.g. data privacy, use and storage) of the iCCAS tool could reduce such uncertainties. At the same time, finding resolution for public concerns about information privacy is an ongoing effort across all eHealth initiatives, and requires proactive efforts to build trust building alongside advances in technology, interoperability, standards and protocols [61]. Providers in our study also expressed a desire to have iCCAS available in different languages to overcome linguistic barriers of the populations served by the Community Health Centres. They also suggested linking the iCCAS generated reports with the client EMR system to optimize its accessibility and efficacy. Further, providers perceived that the use of iCCAS during clients’ initial visits to the Centre, as well as during yearly wellness assessments, could optimize its benefits. Future work should focus on issues of tailoring, scaling up, and sustainability.

Study limitations should be acknowledged to assist in interpretation of the findings. The survey-based assessment of client acceptance of the tool focused on cognitive and technical sides of their experiences. However, acceptance has several other dimensions, such as quality and satisfaction, and future work should include in-depth qualitative interviews with clients to capture the full breadth of acceptance. The qualitative interviews were conducted with nine providers who agreed to participate in the randomized trial. This might be considered a small sample, but it comprised of all of the NPs and FPs at the collaborating clinic. Nonetheless, the transferability of the study findings warrant caution as it was conducted at a single Community Health Centre that primarily serves newcomers, immigrants and refugees from racialized and low-income communities in a North American metropolitan city within a system of government-sponsored health insurance. The Centre staffs an interdisciplinary health care team and is well-resourced for mental health care with a tradition of prioritizing access to these services. Thus, careful consideration of the context is needed in applications to other settings. Finally, future use and evaluation of iCCAS at multiple settings and by diverse teams is encouraged to advance understanding and applicability.

## Conclusions

In conclusion, iCCAS-facilitated visits became patient-centered by bringing clients’ mental health needs to the forefront through patient activation and provider enablement. Both providers and clients found the tool generally acceptable and beneficial for use in the Community Health Centre, suggesting feasibility to incorporate it into other similar practices. When offered through Community Health Centres, the iCCAS technology holds a unique potential to address health disparities by enhancing timely access to care for marginalized ethno-racial and immigrant groups. Technological innovations of iCCAS that helped to catalyze these benefits include the anonymous and personalized feeling of the tool (tablet device that clients hold in their hands and use in private ways or spaces) combined with user-friendly functions (touch-screen entry, with options to go back and forth aided by easy navigational pointers and explanations of questions being asked) and client-centered features (translation into first language for Spanish-speaking clients, questions regarding social determinants and other indicators pertinent to vulnerable clients, and tailored reports at the point of care).

The study findings are likely to help development of future eHealth interventions for mental health screening in primary care. On one side, the burden of mental health is growing globally, and CMDs have a disproportionate impact on low-income, immigrant, and ethnically marginalized groups. On the other side, e-Health innovations are viewed as an essential element of healthcare renewal [32, 62, 63]. The time is right to fuse the two, though implementation challenges are substantial and require design flexibility and use of formative and summative evaluations.

## Abbreviations

AUDIT-C: Alcohol use disorder identification test-C
CAGE: Cut-annoyed-guilty-eye scale
CaPRA: Computer-assisted psychosocial risk assessment
CMD: Common mental disorder
DAST-10: Drug abuse screening test-10
eCHAT: Electronic case-finding and help assessment tool
EMR: Electronic medical record
FP: Family physician
GAD-7: Generalized anxiety disorder-7
iCCAS: Interactive computer-assisted client assessment survey
MOHR: My own health report
NP: Nurse practitioner
PHQ-9: Patient health questionnaire-9
PTSD-PC: Post traumatic stress disorder-primary care
RCT: Randomized controlled trial
RE-AIM: Reach, effectiveness, adoption, implementation and maintenance
SD: Standard deviation
